# miR-324-3p promotes gastric cancer development by activating Smad4-mediated Wnt/beta-catenin signaling pathway

**DOI:** 10.1007/s00535-017-1408-0

**Published:** 2017-11-04

**Authors:** Guang-Li Sun, Zheng Li, Wei-Zhi Wang, Zheng Chen, Lei Zhang, Qing Li, Song Wei, Bo-Wen Li, Jiang-Hao Xu, Liang Chen, Zhong-Yuan He, Kai Ying, Xuan Zhang, Hao Xu, Dian-Cai Zhang, Ze-Kuan Xu

**Affiliations:** 1 Department of General Surgery, The First Affiliated Hospital of Nanjing Medical University, No. 300 Guangzhou road, Nanjing, Jiangsu China; 2 Department of Surgery, Vanderbilt University Medical Center, Nashville, TN USA

**Keywords:** Gastric cancer, miR-324-3p, Smad4, Wnt, Organoid

## Abstract

**Background:**

Emerging evidence suggested that miRNAs can function as oncogenes or tumor suppressors by regulating downstream target genes. miR-324-3p has been reported to function in several carcinomas, but its role in gastric cancer (GC) is still unknown. This study aims to explore the effects of miR-324-3p on the development of GC.

**Methods:**

Expression of miR-324-3p was examined in GC cells and tissues by qRT-PCR. Effects of miR-324-3p on GC cells were evaluated by cell vitality assay, colony formation assay, cell migration assay, and flow cytometric assay. The dual luciferase assay was used to verify whether miR-324-3p could interact with the potential target genes. Western blot was used to assess the expression level of Smad4 and beta-catenin. Intracellular ATP level was also examined. The tumor xenografts were established using nude mice. A gastric organoid model was made from fresh stomach tissue.

**Results:**

miR-324-3p was expressed at higher levels in the tumor tissues compared with adjacent normal tissues. Overexpression of miR-324-3p promoted cell growth, migration, and decreased apoptosis. miR-324-3p repressed the expression of Smad4, and loss of Smad4 activated the Wnt/beta-catenin signaling pathway. Overexpression of Smad4 rescued the effects of miR-324-3p on GC cells. The intracellular ATP level was upregulated with overexpression of miR-324-3p. miR-324-3p facilitated tumor cell colonization and growth in vivo and contributed to the growth of gastric organoids.

**Conclusions:**

The results suggested that miR-324-3p promoted GC through activating the Smad4-mediated Wnt/beta-catenin signaling pathway. The miR-324-3p/Smad4/Wnt signaling axis may be a potential therapeutic target to prevent GC progression.

## Introduction

Gastric cancer (GC) is the fourth most common carcinoma in men and the fifth in women and is the second leading cause of cancer-related death [1]. Overall, 43% of the GC patients are in China [2]. Most of the patients with gastric cancer are diagnosed at an advanced stage and they have a poor prognosis with low 5-year survival rate [3]. One of the reasons is the lack of effective early diagnostic biomarkers. It is necessary to study the molecular mechanism of gastric cancer to determine biomarkers for early diagnosis and novel targets for more effective therapy.

Increasing evidence demonstrates that micro-RNAs (miRNAs) act either as oncogenes or as tumor suppressors in the development and progression of tumors [4]. miRNAs are small, non-coding RNAs that bind to the 3′-untranslational regions (3′-UTRs) of target mRNAs [5, 6]. The target genes usually play a critical role in controlling cancer-related cellular processes such as proliferation, apoptosis, migration, differentiation, and cell cycle progression [7–9].

It has been reported that miR-324-3p was significantly upregulated in plasma of stage I lung squamous cell carcinoma compared to healthy controls [10]. Plasma miR-324-3p level was significantly increased in hepatocellular carcinoma, so it might act as an early biomarker for hepatocellular carcinoma [11]. Previous studies have shown that miR-324-3p acted as a tumor suppressor in nasopharyngeal carcinoma [12]. The effect of miR-324-3p on cancer is still uncertain and the relationship between miR-324-3p and GC remains blank. Whether miR-324-3p could regulate the biological functions of GC cells and the mechanism needs to be explored.

It has been reported that Smad4 was inactivated in different types of carcinomas and acted as a tumor suppressor in GC [13, 14]. Smad4 has been confirmed to suppress Wnt/beta-catenin signaling activity in colon carcinoma [15]. The Wnt/beta-catenin signaling pathway is a highly conserved system during evolution [16]. It has been reported to regulate various processes that are important for cancer progression, such as tumor initiation, tumor growth, cell death, cell senescence, differentiation, and metastasis [17]. In our study, we discovered that Smad4 is one of the targets of miR-324-3p and Smad4-mediated Wnt/beta-catenin signaling activity is activated in GC.

## Methods

### Tissue samples

The 68 pairs of tumor and adjacent normal tissues used in our study were collected from the Department of General Surgery of the First Affiliated Hospital of Nanjing Medical University. None of the patients recruited to this study received any preoperative treatments. Written informed consents were obtained from the patients. No researching processes were undertaken without the informed contents. Our study was approved by the First Affiliated Hospital of Nanjing Medical University Ethics Committee. All specimens were stored in liquid nitrogen before RNA extraction and qRT-PCR analysis. GC patients were staged according to the 7th edition of the American Joint Committee on Cancer (AJCC) tumor node metastasis (TNM) staging system.

### Cell lines and cell culture

Four human gastric cancer cell lines were used in our research, namely MGC-803, BGC-823, HGC-27, and SGC-7901. All four cell lines were purchased from Shanghai Institute for Biological Sciences, Chinese Academy of Sciences. Human normal gastric epithelial cell line (GES-1) was purchased from the American Type Culture Collection. The cell lines were cultured in RPMI1640 (Gibco, Carlsbad, CA, USA) containing 10% fetal bovine serum (FBS, Gibco, Uruguay). All media were supplemented with 100 U/ml penicillin and 100 µg/ml streptomycin (Invitrogen life Technologies, Carlsbad, CA, USA). Cells were maintained in a humidified incubator at 37 °C with 5% CO_2_.

### Quantitative real-time polymerase chain reaction (qRT-PCR)

Total RNA was extracted from frozen tissues and cultured cells with miRNeasy Kit (Qiagen, Dusseldorf, Germany) following the manufacturer’s protocol. We carried out reverse transcription using the Thermo Scientific RevertAid Transcriptase Kit (Thermo, Waltham, MA, USA) on the basis of the manufacturer’s protocol. We used Primescript RT Reagent (Takara, Japan) for mRNA reverse transcription. All the primers (Realgene, Nanjing, China) are listed below: hsa-miR-324-3p forward, 5′-ACTGCCCCAGGTGCTGCTGG-3′; Universal, 5′-GCGAGCACAGAATTAATACGAC-3′; U6 forward, 5′-CTCGCTTCGGCAGCACA-3′; U6 reverse, 5′-AACGCTTCACGAATTTGCGT-3′; Smad4 forward, 5′-GTGACGTTTGGGTCAGGTGC-3′; Smad4 reverse, 5′-TATGAACAGCGTCGCCAGGT-3′; beta-actin forward, 5′-GCATCGTCACCAACTGGGAC-3′; beta-actin reverse, 5′-ACCTGGCCGTCAGGCAGCTC-3′. miR-324-3p expression levels were normalized to snU6 and the expression of Smad4 was normalized to beta-actin. Relative expression was calculated using the 2^−ΔΔCT^ method. We performed quantitative real-time PCR using an ABI StepOne Plus system with SYBR Green Master Mix (Roche, USA) for miRNA and mRNA detection.

### Plasmid construction

The plasmid for Smad4 was created using pcDNA3.1 (Invitrogen, Carlsbad, CA, USA). According to the base sequence of Smad4, we designed the forward primer (5′-ATCTCGAGGAACAAATGGACAATATGTC-3′) and reverse primer (5′-GCGAATTCGTCTAAAGGTTGTGGGTC-3′). Human genomic DNA was used as a template for PCR amplification and the PCR product was subcloned into pcDNA3.1 expression vector. The plasmid was transfected with lipo2000 (Invitrogen) into cells.

### Cell transfection

Lentivirus vectors were used to establish the stable transfected cell lines. Negative control (NC), miR-324-3p mimics, and miR-324-3p inhibitor constructed in lentivirus vectors were purchased from GenePharma (Shanghai, China). We performed the cell transfection following the manufacturer’s protocol.

### Cell proliferation and vitality assay

Cell counting kit-8 (CCK-8, Dojindo, Kumamoto, Japan) was used for these two assays. For the proliferation assay, we first seeded stable transfected cells into a 96-well plate with 2000 cells per well. These cells were incubated for 5 days. We added CCK-8 reagent into each well and incubated for 2 h before measurement every day. For the cell vitality assay, stable transfected cells were seeded into a 96-well plate with 5000 cells per well. After the cells were incubated for 2 days, we measured the absorbance. All the steps were carried out according to the manufacturer’s protocol.

### Colony formation assay

Stable transfected cells were transferred to 6-well plates with 1000 cells per well. The cells were incubated for 3 weeks before being fixed with 75% alcohol and stained with crystal violet. After the cells were washed with phosphate buffered solution (PBS), the number of colonies was counted.

### Transwell migration assay

Cell migration was determined using 24-well BioCoat Matrigel Invasion Chambers (BD Biosciences, Franklin Lakes, NJ, USA). First, we seeded 2 × 10^4^ stable transfected cells onto the upper side of the membrane with 200 µl RPMI 1640 without fetal bovine serum. Then we added 500 µl RPMI 1640 with 10% FBS into the 24-well plate as chemoattractant. After incubating for 24 h, some of the cells migrated to the lower side of the membrane. Cells that did not migrate through the pores were removed with a cotton swab. Finally, we used 75% alcohol to fix the cells and crystal violet to stain the cells. After these steps, we counted the cells that migrated to the other side of the membrane.

### Flow cytometric analysis

We seeded stable transfected cells into a 6-well plate at a density of 2 × 10^5^ per well and the cells were incubated for 2 days. All the cells in each well were collected and stained with a PE Annexin V Apoptosis Detection Kit (BD Pharmingen, Franklin Lakes, NJ, USA). The ratio of the apoptosis cells was detected by flow cytometry. The data was analyzed by CELL Quest software (BD, Biosciences, San Jose, CA, USA).

### Intracellular ATP determination

Stable transfected cells were seeded into opaque-walled 96-well plates at 5000 cells per well. The ATP levels were measured with an ATP Assay Kit (Beyotime) according to the manufacturer’s protocol. We also cultured stable transfected cells with 5% CO_2_, 1% O_2_, and 94% N_2_ in a hypoxic chamber (Invivo200, UK) for intracellular ATP measurement under hypoxic conditions.

### Western blot analysis

Anti-Smad4, anti-beta-catenin, and anti-GAPDH antibodies were purchased from Cell Signal Technology (Boston, MA, USA). Anti-rabbit IgG-HRP and anti-mouse IgG-HPR antibodies were purchased from Santa Cruz (Dallas, TX, USA). Stable transfected cells were lysed with Lysis buffer (Beyotime). A protein extraction kit (Key Gene, Nanjing, China) was used to extract protein from stable transfected cells on the basis of the manufacturer’s protocol. Whole-cell lysate was separated by electrophoresis in SDS-containing polyacrylamide gels and transferred to polyvinylidene fluoride (PVDF) membrane (Millipore, Billerica, MA, USA). The membranes were blocked in TBST buffer containing 5% nonfat dry milk for 2 h and then incubated with primary antibodies as described before overnight at 4 °C. The membranes were washed using TBST buffer three times, each time lasting 10 min. We used the corresponding HRP-labeled secondary antibodies to incubate the membranes for 2 h. Before detection, the membranes were washed with TBST buffer three times. Finally, the blot signals were visualized with the Chemiluminescence HRP Substrate (Millipore, WBKL0100) and an enhanced chemiluminescence detection system.

### Luciferase reporter assay

The potential binding site of miR-324-3p at the 3′-UTR of Smad4 mRNA was computationally predicted by Targetscan. The 3′-UTR sequences of Smad4 containing wild-type (wt) or mutant (mut) miR-324-3p binding sites were synthesized by Genescript (Nanjing, China) and cloned into pGL-3 luciferase reporter vector. The luciferase reporter vectors were co-transfected with MGC-803 with miR-324-3p mimics and NC and BGC-823 with miR-324-3p inhibitor and NC. Luciferase activity was detected by the Dual-Luciferase Reporter Assay System (Promega, Madison, WI, USA). The firefly luciferase activity was normalized to renilla luciferase activity.

TOPflash/FOPflash reporters were purchased from Upstate Biotechnology Inc (Lake Placid, NY, USA). TOPflash and FOPflash reporter plasmids were transfected into cells with Lipofectamine 3000 (Invitrogen). A Dual-Glo Luciferase Assay Kit (Promega) was adopted after 48 h transfection. The activity of firefly luciferase was normalized to that of renilla luciferase.

### Subcutaneous tumor growth assay

The 5-week-old male nude mice (BALB/c nude mice) used in our study were purchased from the Department of Laboratory Animal Center of Nanjing Medical University. All the animal experiments were approved by Nanjing Medical University Ethics Committee (permission number 2014-SR-007). Control and manipulated cells were separately ejected. We injected 1 × 10^6^ stable transfected cells suspended with 100 µl PBS subcutaneously into the flank of nude mice. Nude mice were killed on day 24 and the subcutaneous tumors were removed. Tumor volume was measured on the basis of the following formula: volume = 1/2 × length × width^2^.

### Construction of human gastric organoid model

We constructed a gastric organoid model based on the protocols published previously [18]. Fresh stomach tissues collected from patients were used instead of murine stomach tissue to establish human gastric organoids. We took photos of the gastric organoids every day. The organoids were transfected with miR-324-3p mimics and NC lentivirus. After 10 days of culture, gastric organoids were harvested from Matrigel. After being fixed with 70% ethanol, organoids were made into paraffin sections for immunochemical staining.

### Immunochemical staining

Nude mice subcutaneous tumors, tumor, and adjacent normal tissue collected from patients and gastric organoids were fixed with 4% formaldehyde and then embedded in paraffin. The paraffin mass was cut into 4-µm slices. The slices were incubated with anti-ki67 antibody (Abcam, UK) and anti-Smad4 antibody (Abcam, UK) overnight at 4 °C in a humidified chamber. The slices were washed with PBS three times and incubated with HRP-polymer-conjugated secondary antibody at room temperature for 1 h. 3,3′-Diaminobenzidine (DAB) solution was used to dye the slices for 3 min and hematoxylin was used for counterstaining. Photos of three random fields for each slide were taken and the percentage of positive cells was determined.

### TUNEL assay

The Cell Death Detection Kit (Roche, USA) was used in this assay. The slides were prepared from paraffin mass. Gradient concentration of ethanol was used to rehydrate the slides. After being fixed in 4% formaldehyde, the slides were incubated with proteinase K at room temperature for 15 min. We used 3% hydrogen peroxide to block endogenous peroxidases. TUNEL solution buffer with TdT enzyme was prepared on the basis of the manufacturer’s protocol. Hematoxylin was adopted to stain the slides washed with PBS. The percentage of TUNEL-positive cells was determined with the help of a microscope (Nikon, Japan).

### Statistical analysis

Statistical Product and Service Solutions (SPSS) software version 19.0 was adopted for statistical analysis. All the experiments were performed at least three times. All data is shown as mean ± standard deviation (SD). Student’s *t* test and Pearson *χ* ^2^ test were used in data analysis. *P < 0.05 and **P < 0.01 were considered to indicate statistical significance.

## Results

### miR-324-3p was upregulated in gastric cancer tissues and cells

**Fig. 1:**
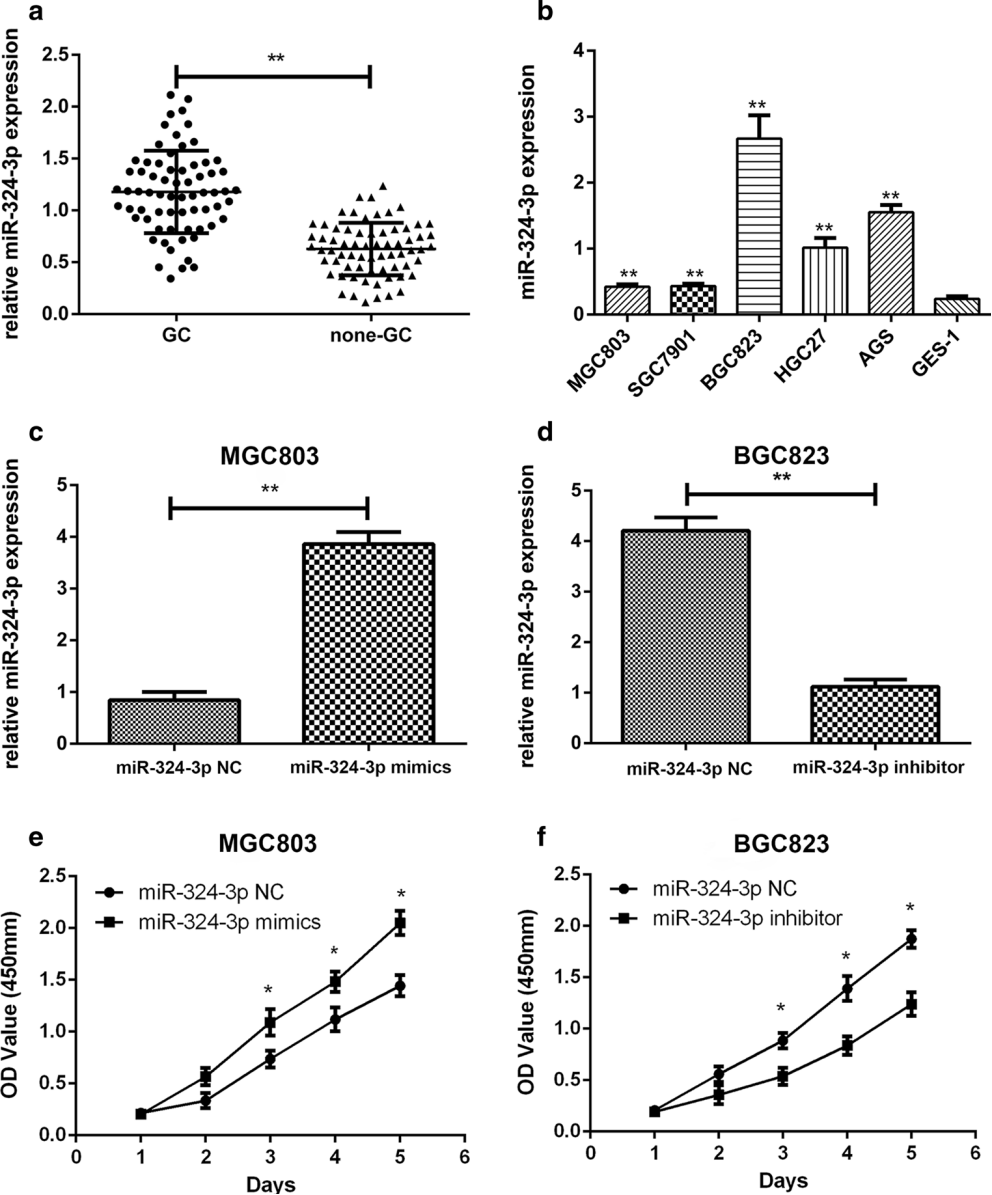
Expression of miR-324-3p in GC tissues, cell lines, and transfected GC cells. **a** miR-324-3p expression was detected in 68 pairs of GC and adjacent normal tissues by qRT-PCR. **b** The expression of miR-324-3p in GC cell lines and GES-1 cell line. **c**, **d** miR-324-3p expression level in GC cell lines transfected with lentivirus miR-324-3p mimics and miR-324-3p inhibitor. **e**, **f** Proliferation rate of transfected GC cells was determined by CCK-8 assay

A total of 68 pairs of GC tumor tissues and adjacent normal tissues were collected to explore the expression of miR-324-3p. As shown in Fig. 1a, the expression of miR-324-3p was higher in tumor tissues than adjacent normal tissues. We then detected the expression of miR-324-3p in GC cells and GES-1 cells. As shown in Fig. 1b, compared with GES-1, the expression level of miR-324-3p was much higher in GC cells. Furthermore, we analyzed the relationship between the expression level of miR-324-3p and the clinicopathological features of the patients. The 68 patients were divided into two groups based on the expression level of miR-324-3p. As shown in Table 1, there was a positive correlation between the tumor size and the expression level of miR-324-3p.

**Table 1:** Expression of miR-324-3p in human gastric cancer and the clinicopathological characteristics of the patients

Characteristics	Number (%)	miR-324-3p expression		*P* value	Smad4 expression		*P* value
		High group	Low group		High group	Low group	
Age (years)							
≥ 60	43 (63.2%)	23	20	0.451	21	22	0.801
< 60	25 (36.8%)	11	14		13	12	
Gender							
Male	37 (54.4%)	18	19	0.808	20	17	0.465
Female	31 (45.6%)	16	15		14	17	
Size (cm)							
≥ 3 (cm)	36 (52.9%)	23	13	0.015*	12	24	0.004*
< 3 (cm)	32 (47.1%)	11	21		22	10	
Stages							
I/II	30 (44.1%)	14	16	0.625	18	12	0.143
III/IV	38 (55.9%)	20	18		16	22	
T grade							
T_1_ + T_2_	38 (55.9%)	17	21	0.329	20	18	0.625
T_3_ + T_4_	30 (44.1%)	17	13		14	16	
Lymph node metastasis							
N1–N3	40 (58.8%)	22	18	0.324	17	23	0.139
N0	28 (41.1%)	12	16		17	11	
Lauren classification							
Intestinal type	30 (44.1%)	12	18	0.330	19	11	0.094
Diffuse type	27 (39.7%)	16	11		12	15	
Mixed type	11 (16.2%)	6	5		3	8	

*P < 0.05 statistically significant difference

### miR-324-3p promoted gastric cancer cell proliferation, migration, and vitality

**Fig. 2:**
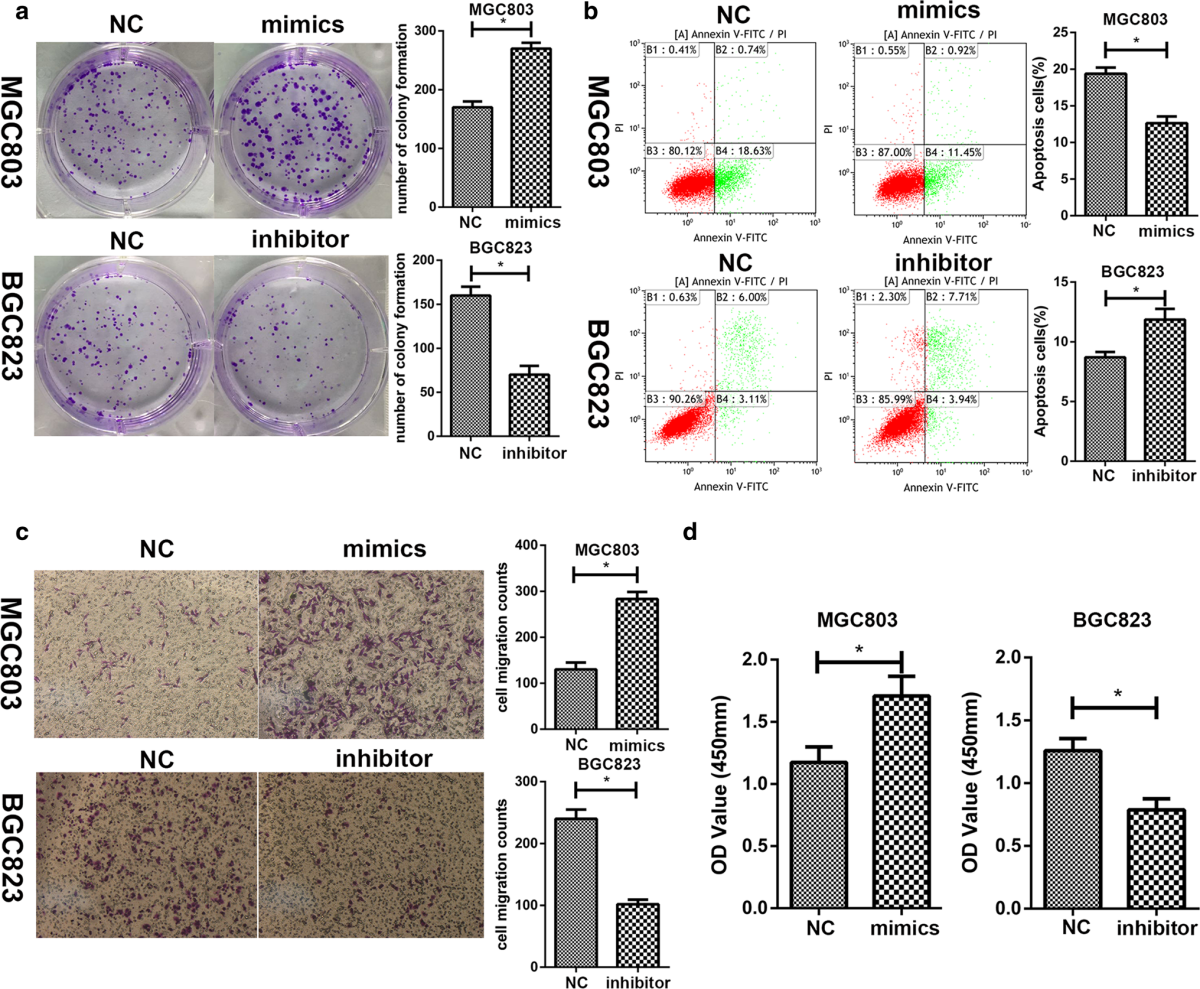
miR-324-3p promoted cell proliferation and inhibited cell apoptosis. **a** miR-324-3p could promote cell proliferation by colony formation assay. **b** The effect of miR-324-3p on cell apoptosis by flow cytometric analysis. **c** miR-324-3p improved cell migration by transwell migration assay. **d** Cell vitality detected by CCK-8 assay on GC cells transfected with miR-324-3p mimics or miR-324-3p inhibitor lentivirus

On the basis of the expression level of miR-324-3p in GC cell lines, we transfected MGC-803 with miR-324-3p mimics and BGC-823 with miR-324-3p inhibitor. To determine the efficiency of lentivirus transfection, we performed qRT-PCR on stable transfected cell lines. The results are shown in Fig. 1c, d. The CCK-8 assay was carried out to explore the effect of miR-324-3p on the proliferation of BGC-823 and MGC-803. As shown in Fig. 1e, f, the proliferation rate of MGC-803 mimics was much higher than the control group MGC-803-NC, while the growth rate of BGC-823 inhibitor was significantly decreased compared with the control group BGC-823-NC. In the colony formation assay, we could see that overexpression of miR-324-3p promoted proliferation of GC cells, while the knockdown of miR-324-3p had the opposite effect (Fig. 2a). We performed flow cytometric analysis to explore how miR-324-3p affected cell apoptosis. As shown in Fig. 2b, miR-324-3p played an inhibitory role in cell apoptosis. To determine the effect of miR-324-3p on the migration of GC cells, we performed a transwell migration assay. In Fig. 2c, miR-324-3p promoted GC cell migration, and blocking miR-324-3p inhibited GC cell migration. To explore the relationship between miR-324-3p and the cell vitality, the CCK-8 cell vitality assay was performed. The cell vitality of MGC-803 mimic cells was much higher than that of MGC-803-NC cells, while the cell vitality of BGC-823 was decreased after transfection of miR-324-3p inhibitor (Fig. 2d).

### Smad4 is a direct target of miR-324-3p

**Fig. 3:**
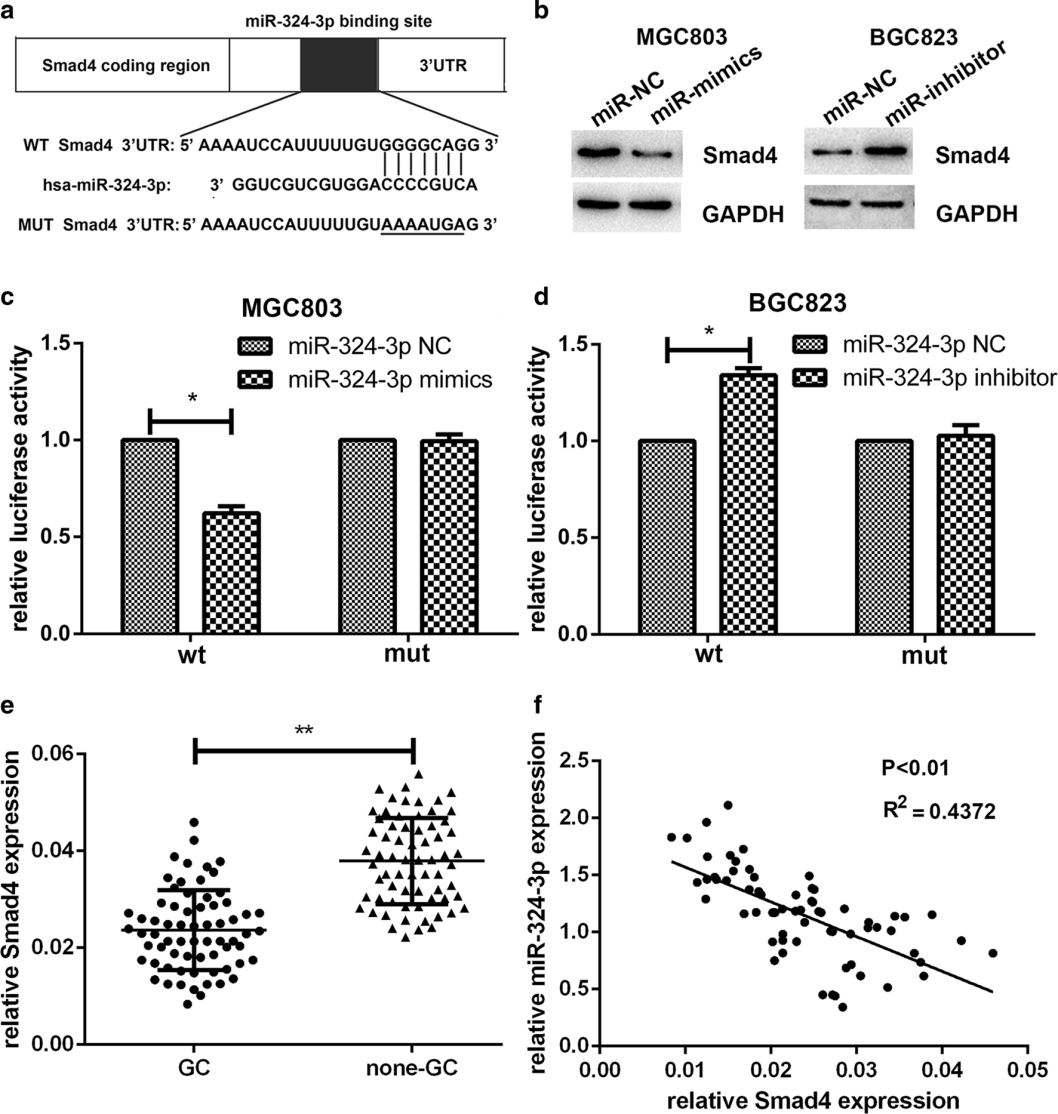
Smad4 was a direct target of miR-324-3p. **a** The predicted binding site of miR-324-3p at the 3′UTR of Smad4 by Targetscan. **b** Effect of miR-324-3p on the expression level of Smad4 by western blot. **c** Luciferase activity analyzed with MGC-803 cells co-transfected with mimics or NC and pGL3-Smad4 or pGL3-Smad4-mut. **d** Luciferase activity analyzed with BGC-823 cells co-transfected with inhibitor or NC and pGL3-Smad4 or pGL3-Smad4-mut. **e** Expression level of Smad4 in 68 pairs of GC tissues and adjacent normal tissues was detected by qRT-PCR. **f** There was a negative correlation between the expression level of miR-324-3p and Smad4 in 68 pairs of GC tissues

The effect of miR-324-3p on biological functions of GC cells showed that miR-324-3p plays a promotive role in gastric cancer. miRNAs are mainly negative regulators of mRNA translation [19], so the targets of miR-324-3p are likely be tumor suppressors. Smad4 has been reported to suppress gastric cancer and be involved in modulating cell proliferation, apoptosis, and migration [14, 20]. On the basis of the prediction of miRanda (http://www.microrna.org/microrna/home.do) and Targetscan (http://www.targetscan.org/) and the effect upon GC, Smad4 was found to be a potential target of miR-324-3p (Fig. 3a). Western blot was used to detect the change of Smad4 expression after lentivirus transfection. As shown in Fig. 3b, overexpression of miR-324-3p decreased the expression of Smad4, whereas blocking of miR-324-3p upregulated Smad4 expression. To demonstrate that Smad4 was a direct target of miR-324-3p, the dual-luciferase reporter assay was conducted. We observed that co-transfection with miR-324-3p mimics and pGL3-Smad4 vector displayed an obvious reduced luciferase activity in MGC-803. It was also noticed that co-transfection with miR-324-3p inhibitor and pGL-Smad4 vector showed an increased luciferase activity in BGC-823. However, the luciferase activity was not affected in cells co-transfected with miR-324-3p mimics or inhibitor and pGL3-Smad4-mut vector (Fig. 3c, d). To explore the expression level of Smad4 in gastric cancer tissues and adjacent normal tissues, we performed qRT-PCR on 68 patient samples and the results are shown in Fig. 3e. Furthermore, we analyzed the correlation between Smad4 expression and the clinicopathological features of the 68 GC patients. As shown in Table 1, Smad4 expression showed a negative correlation with tumor size. We also noticed that there was a negative correlation between the expression level of miR-324-3p and Smad4 (Fig. 3f). These results suggested that Smad4 could be one of the direct targets of miR-324-3p.

### Smad4 could reverse the effects of miR-324-3p mimics on gastric cancer cells

**Fig. 4:**
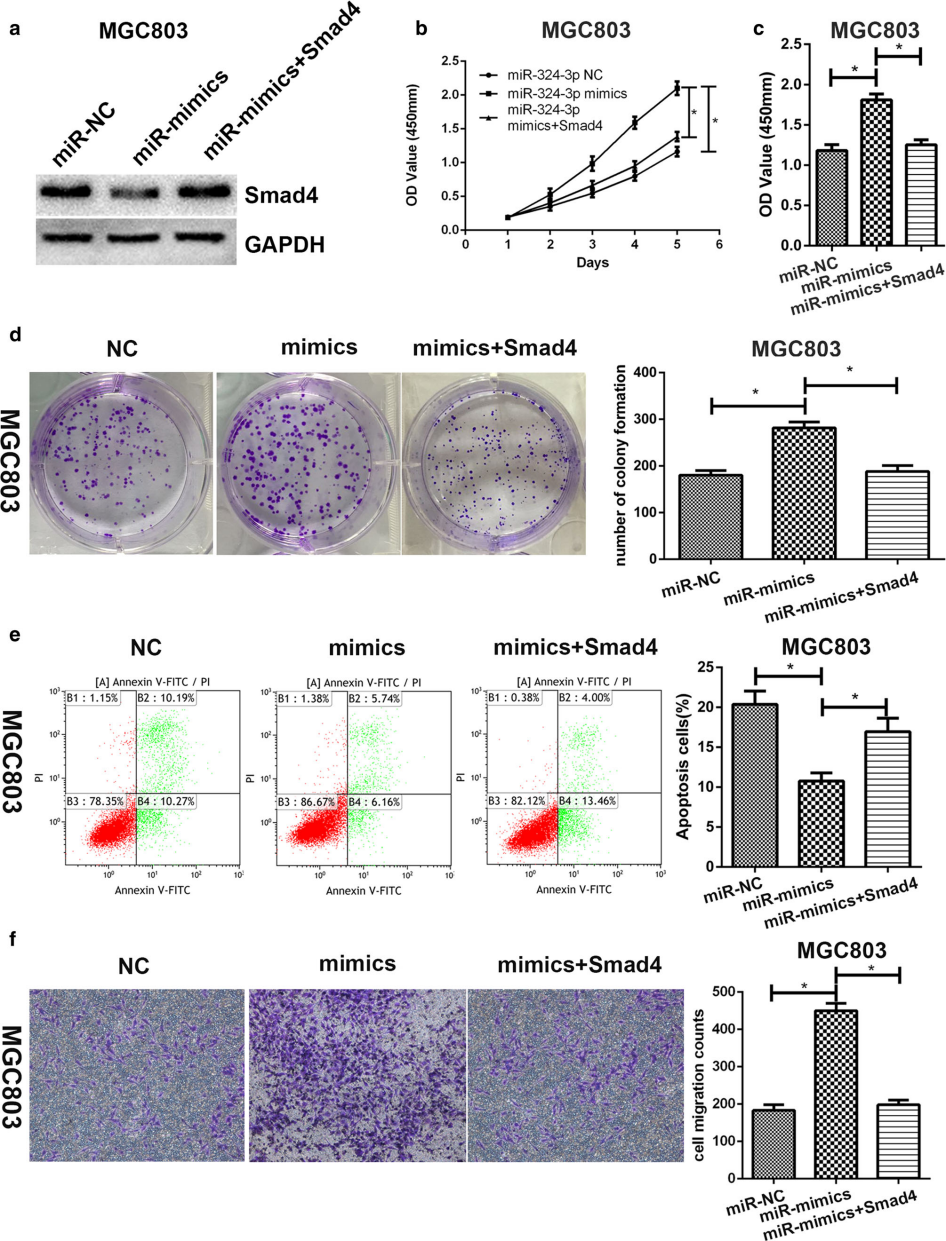
Overexpression of Smad4 could reverse the effect of miR-324-3p on GC cells. **a** Smad4 protein level was detected by western blot. **b** Smad4 could reverse the effect of miR-324-3p on cell proliferation. **c** Smad4 could reverse the effect of miR-324-3p on cell vitality. **d** Restoration of Smad4 inhibited colony formation. **e** Flow cytometric assay was performed to verify that Smad4 could reverse the effect of miR-324-3p on cell apoptosis. **f** Transwell migration assay demonstrated that cell migration ability was restrained by overexpression of Smad4

MGC-803 mimic cell line was transfected with pcDNA3.1-Smad4. Then, we performed western blot to make sure the expression level of Smad4 was increased (Fig. 4a). Cell proliferation assay, cell vitality assay, colony formation assay, transwell migration assay and flow cytometric assay were carried out to determine whether overexpression of Smad4 could reverse the effects of miR-324-3p mimics. We noticed that the proliferation rate was reduced by overexpression of Smad4 (Fig. 4b). It was also observed that overexpression of Smad4 inhibited cell vitality (Fig. 4c). As shown in Fig. 4d, e, overexpression of Smad4 could reverse the effects of miR-324-3p on colony formation and cell apoptosis. Increased cell migration caused by miR-324-3p overexpression was reversed by Smad4 (Fig. 4f). To sum up, overexpression of Smad4 could counteract the effect of miR-324-3p on GC cell lines.

### miR-324-3p promoted tumorigenesis, proliferation, and inhibited cell apoptosis of gastric cancer cells in vivo through downregulation of Smad4

**Fig. 5:**
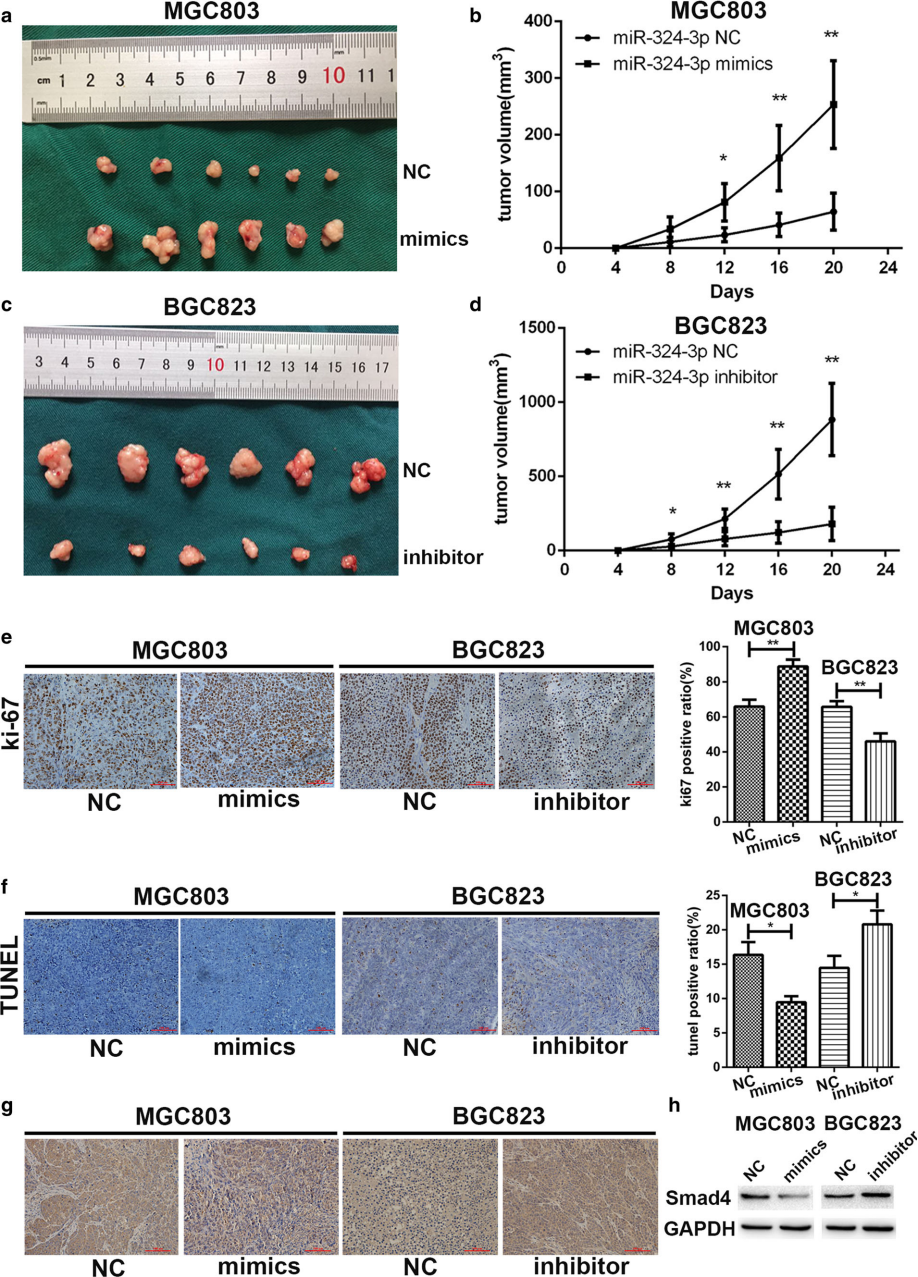
miR-324-3p promoted growth of GC cells in vivo. **a**, **b** Nude mice injected subcutaneously with transfected GC cells were killed and the tumors of the mimics group had a larger volume. **c**, **d** Tumors of inhibitor group had smaller volume than the NC group. **e** The results of ki-67 staining assay showed that miR-324-3p promoted proliferation of GC cells in nude mice. **f** Overexpression of miR-324-3p could inhibit cell apoptosis while knockdown of miR-324-3p promoted cell apoptosis in vivo by TUNEL assay. **g**, **h** Smad4 expression had a negative correlation with miR-324-3p in vivo by immunohistochemistry staining and western blot

To investigate the influence of miR-324-3p on tumor in vivo, a nude mice xenograft model was established. We injected 1 × 10^6^ MGC-803 mimic cells and MGC-803-NC cells subcutaneously into the flank of nude mice respectively. BGC-823 inhibitor and BGC-823-NC cells were injected subcutaneously into the flank of nude mice as well. The volume of the tumors was measured every 4 days with a caliper. The nude mice were killed on day 24 and tumors were collected. As shown in Fig. 5a, b, tumors in the MGC-803 mimics group had larger volume compared with the negative control group. In contrast with the BGC-823-NC group, the tumors in the BGC-823 inhibitor group showed an obvious decrease in volume (Fig. 5c, d). Then we performed immunochemical staining on the collected tumors with anti-ki67 antibody. In Fig. 5e, we noticed that miR-324-3p could promote cell proliferation (ki-67). TUNEL assay was also performed. Compared with negative control, there were more TUNEL-positive cells in the BGC-823 inhibitor group while there were less TUNEL-positive cells in the MGC-803 mimic group, which suggested that miR-324-3p could inhibit cell apoptosis (Fig. 5f). To detect the expression level of Smad4 in collected tumors, immunochemical staining and western blot were conducted with anti-Smad4 antibody. We found that the expression of Smad4 was downregulated in the MGC-803 mimic group but upregulated in the BGC-823 inhibitor group (Fig. 5g, h).

### miR-324-3p could increase the size of gastric organoid and promote its proliferation rate

**Fig. 6:**
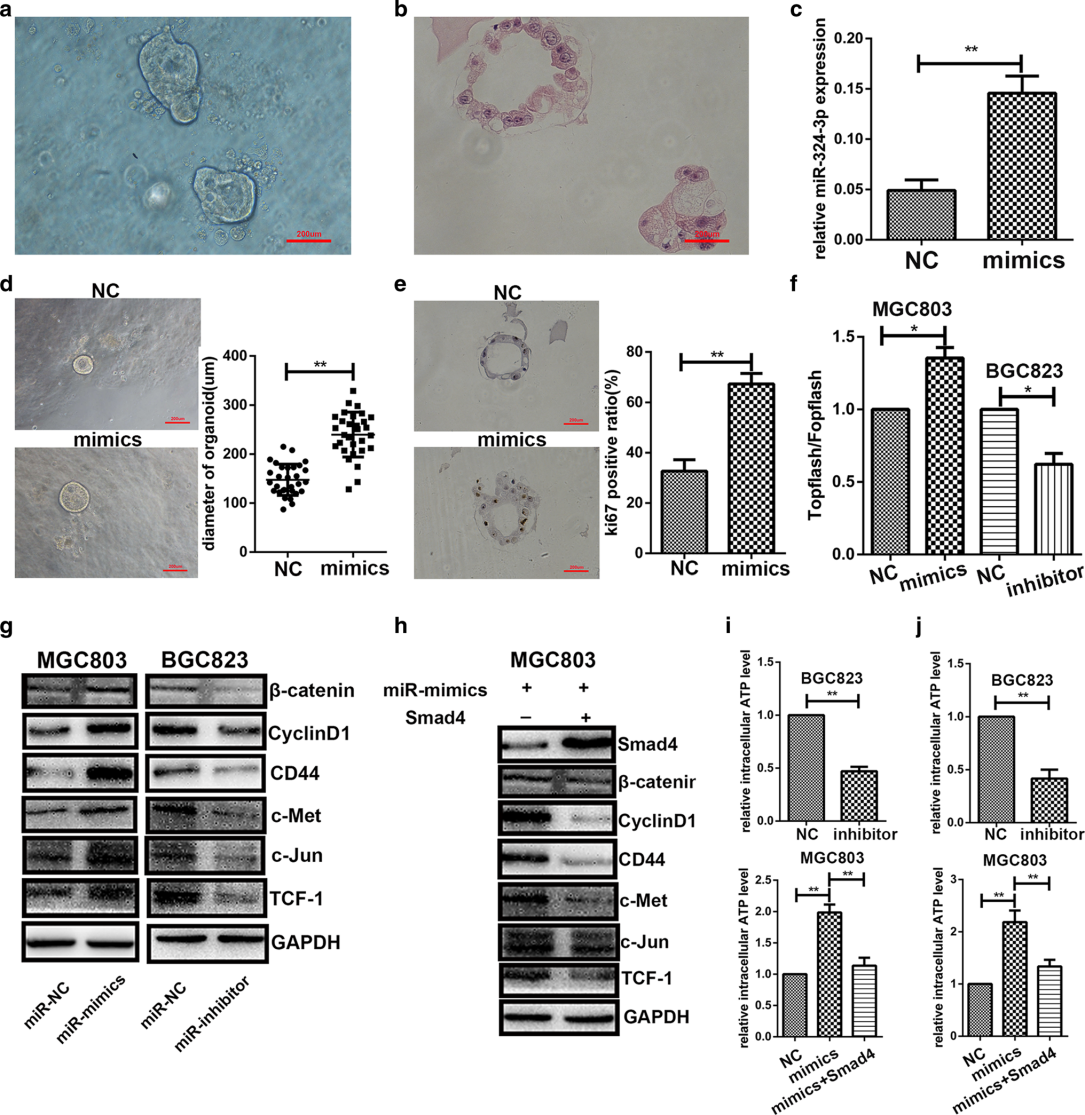
miR-324-3p promoted the growth of gastric organoids and activated the Wnt/beta-catenin signaling pathway via downregulation of Smad4. **a** Photo of gastric organoids we constructed. **b** HE staining of gastric organoids. **c** The expression of miR-324-3p in gastric organoids was upregulated by miR-324-3p mimics lentivirus. **d** miR-324-3p could increase the size of gastric organoids. **e** The results of ki-67 staining showed that miR-324-3p had a positive effect on growth of gastric organoids. **f** The results of TOPflash/FOPflash showed that the Wnt/beta-catenin signaling pathway was activated by overexpression of miR-324-3p and knockdown of miR-324-3p inhibited the Wnt/beta-catenin signaling pathway. **g** Overexpression of miR-324-3p increased the protein expression level of beta-catenin and Wnt-dependent cyclin D1, CD44, c-Met, c-Jun, and TCF-1 while inhibition of miR-324-3p had the opposite effect. **h** Overexpression of Smad4 downregulated the expression level of beta-catenin, cyclin D1, CD44, c-Met, c-Jun, and TCF-1. **i** Knockdown of miR-324-3p reduced intracellular ATP level in the BGC-823 cell line. miR-324-3p increased intracellular ATP level in the MGC-803 cell line and Smad4 could reverse the effect of miR-324-3p on intracellular ATP level. **j** The intracellular ATP levels of GC cells under hypoxic conditions

An organoid is a simplified version of an organ produced in vitro in three dimensions. It enabled us to study diseases directly on human tissue [21]. We constructed gastric organoids using fresh stomach tissue collected from patients (Fig. 6a) and we performed HE staining on it (Fig. 6b). The results of qRT-PCR (Fig. 6c) indicated that the expression level of miR-324-3p was higher in the mimics group compared with the NC group. We measured the diameter of 30 organoids in each group and found that miR-324-3p contributed to the growth of gastric organoids (Fig. 6d). The results of ki-67 staining (Fig. 6e) showed that miR-324-3p could promote the proliferation rate of gastric organoids. miR-324-3p was demonstrated to play a promotive role in gastric cancer with the gastric organoid model.

### miR-324-3p regulated Wnt/beta-catenin signaling pathway through Smad4

It has been reported that Smad4 could regulate the Wnt/beta-catenin signaling pathway [15], so we hypothesized that miR-324-3p functioned as an oncogene through activating the Wnt/beta-catenin signaling pathway. To verify whether the Wnt/beta-catenin signaling pathway was activated, TOPflash/FOPflash luciferase assay was performed on MGC-803 and BGC-823. As shown in Fig. 6f, relative TOPflash/FOPflash luciferase activity was increased by overexpression of miR-324-3p whereas inhibition of miR-324-3p reduced the relative TOPflash/FOPflash luciferase activity, suggesting that miR-324-3p was implicated in Wnt/beta-catenin-dependent transcriptional activity. Then we performed western blot to detect the expression level of beta-catenin and Wnt-dependent genes, such as cyclin D1, CD44, c-jun, c-Met, and TCF-1. From Fig. 6g, we could see that the expression level of these genes was upregulated by overexpression of miR-324-3p while the inhibition of miR-324-3p had the opposite effect. Smad4 was restored in the MGC-803 cell line by transfecting with pcDNA3.1-Smad4. Then western blot was carried out to explore the effect of Smad4 on the Wnt/beta-catenin pathway. As shown in Fig. 6h, the expression level of Smad4 was restored and the restoration of Smad4 reduced the expression level of cyclin D1, CD44, c-jun, c-Met, and TCF-1. To summarize, miR-324-3p might regulate the Wnt/beta-catenin signaling pathway through Smad4.

### miR-324-3p contributed to intracellular ATP generation

A tumor requires a high level of ATP for survival, proliferation, and metastasis [22]. Since we have demonstrated that miR-324-3p could activate the Wnt/beta-catenin signaling pathway and activation of the Wnt/beta-catenin signaling pathway has been reported to increase intracellular ATP level [23], we investigated whether miR-324-3p could add to ATP production to contribute to oncogenesis. In Fig. 6i, we observed that the intracellular ATP level was decreased with knockdown of miR-324-3p in the BGC-823 cell line. It was also observed that miR-324-3p increased the intracellular ATP level and overexpression of Smad4 reversed the effect of miR-324-3p on ATP production in the MGC-803 cell line. As most growing solid tumors contain regions that experience hypoxia [24], and miR-324-3p expression is higher in larger tumors, we also measured intracellular ATP level under hypoxic conditions. The outcomes are shown in Fig. 6j. These results suggested that upregulation of intracellular ATP level caused by miR-324-3p was one of the causes of oncogenesis in GC.

## Discussion

Gastric cancer is a common disease throughout the world, especially in China, and causes hundreds of thousands of deaths every year [1, 2, 25]. Although different therapy methods have been performed, patients diagnosed with advanced GC usually have a poor prognosis [3, 26]. miRNAs are small, non-coding RNAs, acting as oncogenes or tumor suppressors in different types of carcinomas [4, 27]. Many miRNAs have been confirmed to be associated with GC. For example, miR-874 inhibits cell proliferation, migration, and invasion by targeting AQP-3 in gastric cancer [28]. Overexpression of miR-181a-5p promoted the development of GC through activating the RASSF6-mediated MAPK signaling pathway [29]. miR-520b/e could regulate cell proliferation and migration by targeting EGFR in gastric cancer [30]. It has been reported that miR-324-3p was upregulated in the plasma of hepatocellular carcinoma patients [11] and played an inhibitory effect in nasopharyngeal carcinoma [12]. However, the role of miR-324-3p in gastric cancer remains unknown.

In our research, we first explored the expression level of miR-324-3p in 68 pairs of tumor tissues and adjacent non-tumor tissues and found that miR-324-3p expression was higher in GC tissues. The clinicopathological data of the patients was collected and it showed that the expression level of miR-324-3p was related to the tumor size. The GC cell lines also had higher expression levels of miR-324-3p than the GES-1 cell line. Considering that miR-324-3p was upregulated in GC tissues and GC cell lines, we supposed that miR-324-3p might play an oncogenic role in GC. As we speculated, the results of the cell proliferation assay, cell vitality assay, colony formation assay, transwell migration assay, and flow cytometric analysis revealed that miR-324-3p could promote GC.

To further study the mechanism of the biological function of miR-324-3p in GC cells, several databases were used to predict the possible targets of miR-324-3p. Smad4 was predicted to be a possible candidate target. Smad4 is one of the members of the Smad family and is the downstream of TGF-beta signaling pathway. Several pieces of evidence have proved that Smad4 is inactivated in GC and acts as a tumor suppressor in GC [20, 31, 32]. The luciferase reporter assay was performed to confirm that miR-324-3p could bind to the 3′UTR of Smad4 and therefore Smad4 was a direct target of miR-324-3p. The expression level of Smad4 was detected in the 68 pairs of GC tissues and we found that miR-324-3p expression was inversely correlated with Smad4 expression. The clinicopathological data showed that Smad4 expression was negatively correlated with tumor size. We also proved that overexpression of miR-324-3p in GC cell lines could reduce Smad4 protein expression by western blot. To verify whether Smad4 could reverse the effect of miR-324-3p on biological functions of GC cells, pcDNA3.1-Smad4 was transfected into the MGC-803 mimics cell line. As we supposed, restoration of Smad4 inhibited cell proliferation, vitality, and migration. There was enough evidence to demonstrate that Smad4 was a direct target of miR-324-3p.

To study the function of miR-324-3p in GC in vivo, we constructed a tumor xenograft model by injecting GC cells transfected with lentivirus into the flank of nude mice. All the mice were killed on day 24 and we found a positive correlation between the expression level of miR-324-3p and the volume and weight of the tumor. The result of the ki-67 staining showed that miR-324-3p contributed to the proliferation of GC cells in vivo. By TUNEL assay, it was observed that miR-324-3p played an inhibitory role in cell apoptosis in GC cells in vivo. Smad4 expression level was also proved to be negatively correlated with miR-324-3p through immunohistochemistry staining and western blot. Therefore, miR-324-3p could promote GC both in vitro and in vivo.

The Wnt/beta-catenin signaling pathway is highly conserved and aberrant Wnt/beta-catenin signaling pathway activity underlies a variety of pathologies in humans [33]. The Wnt/beta-catenin signaling pathway has been reported to be implicated in GC [34, 35]. Smad4 has been proved to regulate the Wnt/beta-catenin pathway in cranial neural crest cells during tooth morphogenesis [36]. Smad4 has also been demonstrated to suppress the Wnt/beta-catenin pathway in human colon carcinoma cells and pancreatic ductal adenocarcinoma cells [16, 37]. Whether Smad4 could regulate the Wnt/beta-catenin signaling pathway in gastric cancer still remains unknown. We hypothesized that miR-324-3p could activate the Wnt/beta-catenin signaling pathway via loss of Smad4 in GC. TOPflash/FOPflash luciferase assay was conducted to confirm that the Wnt/beta-catenin signaling pathway was activated by overexpression of miR-324-3p. Then we carried out western blot to detect the expression level of beta-catenin and Wnt-dependent genes, such as cyclin D1, CD44, c-jun, c-Met, and TCF-1. As we expected, beta-catenin and Wnt-dependent genes were positively correlated with miR-324-3p. To verify the effect of Smad4 on the Wnt/beta-catenin signaling pathway, western blot was performed on MGC-803 co-transfected with miR-324-3p mimics and pcDNA3.1-Smad4. The results showed that Smad4 could inhibit the Wnt/beta-catenin signaling pathway activated by miR-324-3p.

Organoids could reflect key structural and functional properties of organs so they can be used to model human organ development and various human pathologies [38]. The gastric organoid model used in our research was constructed from fresh stomach tissue collected from patients. The results of ki-67 staining and measurement of size of gastric organoids indicated that miR-324-3p could promote development of gastric organoids. Activation of the Wnt/beta-catenin signaling pathway was reported to be implicated in the development and differentiation of gastric organoid [38, 39]. The Wnt/beta-catenin signaling pathway was proved to be activated by miR-324-3p in our research. miR-324-3p probably promoted gastric organoid development through activation of the Wnt/beta-catenin signaling pathway and further research needs to be done to authenticate this.

Activation of the Wnt/beta-catenin signaling pathway has also been proved to contribute to ATP generation [23]. On the basis of this evidence, we hypothesized that miR-324-3p might add to intracellular ATP level to promote GC. Hypoxia is also involved in tumor metabolism [24], so intracellular ATP measurement was also performed under hypoxic conditions. The results of ATP detection suggested that there was a positive link between the expression level of miR-324-3p and intracellular ATP level. Smad4 was also proved to reverse the effect of miR-324-3p on ATP generation. According to these results, we proved that miR-324-3p could promote ATP production in GC cells.

Our results showed that miR-324-3p activated the Wnt/beta-catenin signaling pathway via downregulation of Smad4. However, we cannot rule out the possibility that there might be other signaling pathways affected by miR-324-3p. Further research needs to be done to study the relationship between miR-324-3p and other signaling pathways. *H. pylori* infection has been reported to be one of the causes of GC [40]. However, most of the 68 patients did not undergo an *H. pylori* examination before, so we failed to assess the relationship between *H. pylori* status and the expression levels of miR-324-3p or Smad4. Whether *H. pylori* infection could affect miR-324-3p or Smad4 expression will be explored in our further research.

## Abbreviations

AJCC: American Joint Committee on Cancer
cck-8: Cell counting kit-8
DAB: 3,3′-Diaminobenzidine
FBS: Fetal bovine serum
GC: Gastric cancer
miRNA: micro-RNA
PBS: Phosphate buffered solution
PCR: Polymerase chain reaction
PVDF: Polyvinylidene fluoride
TNM: Tumor node metastasis
TUNEL: Terminal deoxynucleotidyl transferase-mediated dUTP nick end labeling
3′-UTR: 3′-Untranslational region
